# Web-Based Telepresence Exercise Program for Community-Dwelling Elderly Women With a High Risk of Falling: Randomized Controlled Trial

**DOI:** 10.2196/mhealth.9563

**Published:** 2018-05-28

**Authors:** Jeeyoung Hong, Hyoun-Joong Kong, Hyung-Jin Yoon

**Affiliations:** ^1^ Biomedical Research Institute Seoul National University Hospital Seoul Republic Of Korea; ^2^ Institute of Medical & Biological Engineering Medical Research Center Seoul National University College of Medicine Seoul Republic Of Korea; ^3^ Department of Biomedical Engineering Chungnam National University College of Medicine Daejeon Republic Of Korea; ^4^ Medical Information Center Department of Biomedical Engineering Chungnam National University Hospital Daejeon Republic Of Korea; ^5^ Department of Biomedical Engineering Seoul National University College of Medicine Seoul Republic Of Korea; ^6^ Department of Biomedical Engineering Seoul National University Hospital Seoul Republic Of Korea

**Keywords:** telegeriatrics, resistance exercise, supervised exercise, home exercise, WebRTC, telepresence

## Abstract

**Background:**

While physical exercise is known to help prevent falls in the elderly, bad weather and long distance between the home and place of exercise represent substantial deterrents for the elderly to join or continue attending exercise programs outside their residence. Conventional modalities for home exercise can be helpful but do not offer direct and prompt feedback to the participant, which minimizes the benefit.

**Objective:**

We aimed to develop an elderly-friendly telepresence exercise platform and to evaluate the effects of a 12-week telepresence exercise program on fall-related risk factors in community-dwelling elderly women with a high risk of falling.

**Methods:**

In total, 34 women aged 68-91 years with Fall Risk Assessment scores >14 and no medical contraindication to physical training-based therapy were recruited in person from a senior citizen center. The telepresence exercise platform included a 15-inch tablet computer, custom-made peer-to-peer video conferencing server system, and broadband Internet connectivity. The Web-based program included supervised resistance exercises performed using elastic resistance bands and balance exercise for 20-40 minutes a day, three times a week, for 12 weeks. During the telepresence exercise session, each participant in the intervention group was supervised remotely by a specialized instructor who provided feedback in real time. The women in the control group maintained their lifestyle without any intervention. Fall-related physical factors (body composition and physical function parameters) and psychological factors (Korean Falls Efficacy Scale score, Fear of Falling Questionnaire score) before and after the 12-week interventional period were examined in person by an exercise specialist blinded to the group allocation scheme.

**Results:**

Of the 30 women enrolled, 23 completed the study. Compared to women in the control group (n=13), those in the intervention group (n=10) showed significant improvements on the scores for the chair stand test (95% confidence interval -10.45 to -5.94, *P*<.001), Berg Balance Scale (95% confidence interval -2.31 to -0.28, *P*=.02), and Fear of Falling Questionnaire (95% confidence interval 0.69-3.5, *P*=.01).

**Conclusions:**

The telepresence exercise program had positive effects on fall-related risk factors in community-dwelling elderly women with a high risk of falling. Elderly-friendly telepresence technology for home-based exercises can serve as an effective intervention to improve fall-related physical and psychological factors.

**Trial Registration:**

Clinical Research Information Service KCT0002710; https://cris.nih.go.kr/cris/en/search/ search_result_st01.jsp?seq=11246 (Archived by WebCite at http://www.webcitation.org/6zdSUEsmb)

## Introduction

Falls represent a serious threat to quality of life in the elderly [1]. It has been reported that the yearly incidence of falls among the elderly population aged over 65 years is 30-50% globally [2]. Various factors have been reported to influence the risk of falls among the elderly, including decreasing strength of the lower extremities, cognitive disorders, fear of falling, depression, environmental factors, and problems with walking, balance, vision, feet, or even shoes. Previous studies have shown that there is no single factor responsible for falls, and falling occurs because of complex interactions among multiple factors [3,4]. Nevertheless, the incidence of falls among the elderly can be reduced through preventive activities. Physical exercise has been shown to be very effective in lowering the risk and incidence rate of falls [5]. In particular, resistance exercises are known to be effective at increasing muscle mass, strength, and balance in the elderly [6] and are thus highly indicated to prevent falls in this population. Moreover, balance exercises, such as stepping over a slipper before bending down to pick it up, tandem walking, and side-stepping while holding on to the back of the chair have been shown to be effective in decreasing the risk of subsequent falls [7].

In many countries, including South Korea, elderly people perform unsupervised aerobic exercises in specific places such as senior welfare centers, parks, schoolyards, and public health centers [8]. However, compared to the younger or middle-aged population, the elderly are more affected by weather conditions, as reflected by their lower attendance rate [9]. Moreover, because traveling is typically more difficult for the elderly, the distance between the residence and the place of exercise represents another deterrent for the elderly to join or continue attending exercise programs outside the home. As a result, elderly people are more likely to develop sarcopenia due to the decrease in physical activity, thus increasing their risk of falls [10].

Several solutions have been proposed to overcome the limitations of conventional home exercise, including home fitness equipment, home work-out videos, and fitness apps for mobile phones or computers [11]. However, since these types of unsupervised exercise do not offer direct and prompt feedback to the participant, it is difficult for the elderly to benefit fully from such exercise programs, and the risk of injury is higher. Exergames that employ a three-dimensional depth camera have been introduced over the past few years and have gained attention in the field of physical exercise-based therapy because they can provide real-time feedback to participants using natural user interface technologies [12]. However, most exergames emphasize the fun elements because they target younger age groups; therefore, exergames are of limited use in the elderly population [13]. In addition, in a previous study involving tele-exercise using Skype [14], the user interface and experience were found to not be elderly-friendly. While many technologies for remote supervision are available, such approaches are not readily adopted by elderly people, who generally have a poorer understanding of computers and similar technologies [15].

It is known that falls lead to a fear of falling, depression, and a sense of alienation [16]. The elderly tend to have a passive attitude towards exercising due to fear of falling, as well as depression associated with falling and fall-related injuries. Therefore, this population benefits from steady exercises with interactive feedback, which can help reduce the fear of falling and increase self-confidence related to physical exercise [17]. However, conventional exercise modalities developed for elderly individuals do not provide sufficient fall prevention [18].

In this study, we propose a home-based telepresence exercising program as a new intervention method to prevent falls among high-risk, elderly individuals. Specifically, we developed a novel telepresence exercise platform using information and communication technology to provide supervised physical training sessions to elderly participants at home, with real-time feedback from a remotely located instructor trained to assess the physical, cognitive, and emotional response of the elderly participant. Finally, we investigated the effect of the telepresence training program on fall-related physical and psychological risk factors in home-dwelling elderly women at risk of falls.

## Methods

### Participants

**Figure 1:**
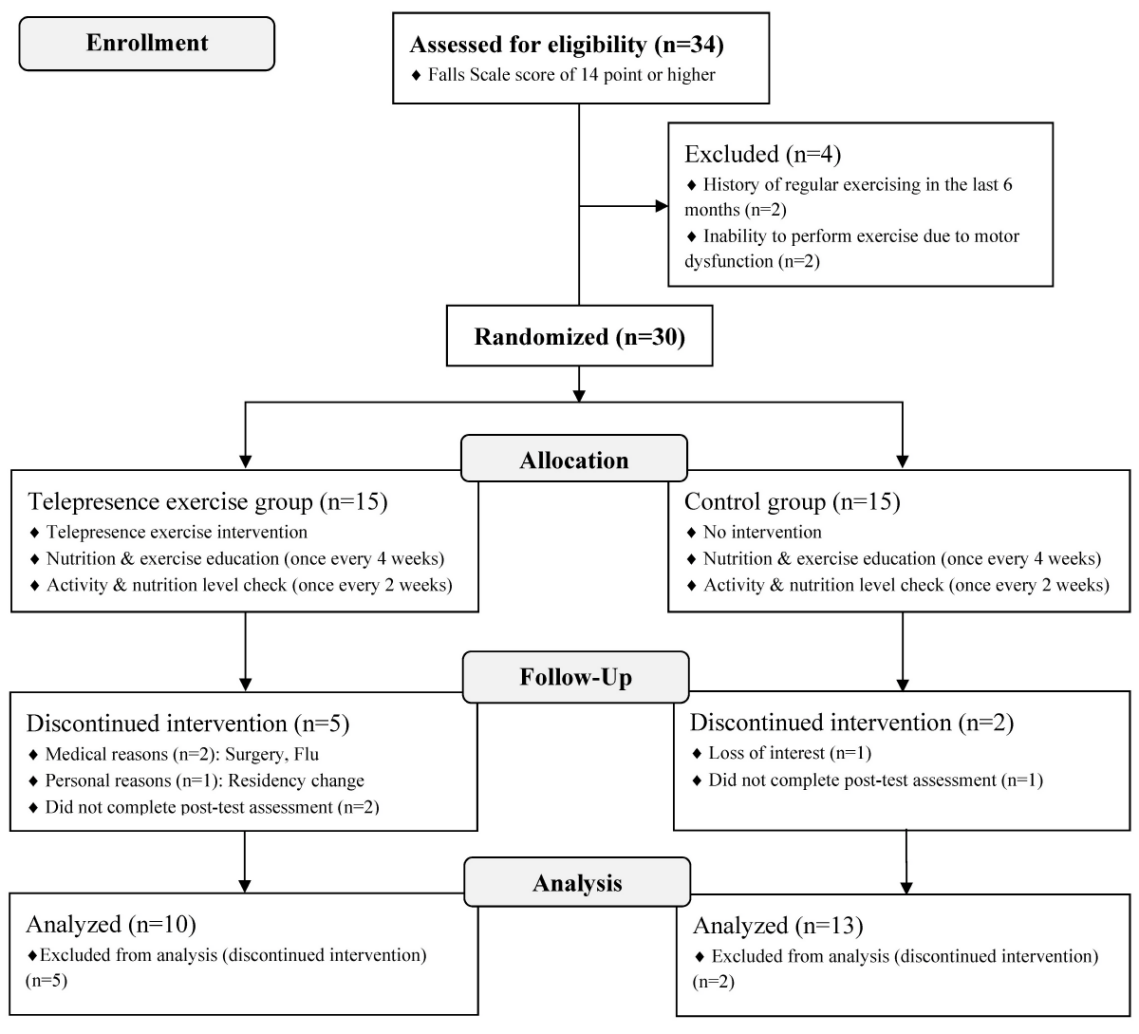
Flow diagram of the study design.

This study was designed as a double-blind, parallel-group, randomized controlled trial. Thirty-four community-dwelling elderly women aged above 65 years and having a Fall Risk Assessment Scale [19] score of 14 points or higher were recruited from the Senior Citizen Centre in Gangseo-gu, Seoul, South Korea, from February 10-March 8, 2015. Recruitment was performed in person by a research coordinator. Computer or Internet literacy was not required as an eligibility criterion. Four women were excluded based on the following criteria: history of regular exercise in the 6 months leading up to the study, inability to exercise due to contraindication to training therapy, or mental illness. The remaining 30 women were assigned to either the telepresence exercise intervention group (IG; n=15) or to the control group (CG; n=15). The group allocation scheme, which was based on simple random sampling performed in Microsoft Excel, was obtained by the research coordinator. Of the 30 women enrolled, 7 discontinued the intervention for medical reasons, did not undergo the posttest assessment, or withdrew from the study due to personal reasons (Figure 1).

The remaining 23 participants completed the study (including the pre- and posttest assessments), and their data were included in the present analysis. Power analysis was performed using G*power 3 [20] and revealed that the sample size of 10 and 13 participants for the IG and CG groups, respectively, was sufficient to distinguish an effect size of 0.35 (for lower body strength) with an alpha value (probability of Type 1 error) of .05 and statistical power (1-beta) of .80. The study was approved by the Seoul National University Hospital Institutional Review Board (Approval No. 1412-150-636) and was registered with the Clinical Research Information Service, which is a primary registry of the World Health Organization International Clinical Trials Registry Platform. All participants provided written informed consent before enrollment and were free to withdraw from the study at any time. The trial is reported in accordance with the CONSORT-EHEALTH provisions for improving and standardizing evaluation reports of Web-based and mobile health interventions (see Multimedia Appendix 1 for the CONSORT-EHEALTH checklist [21]).

### Procedures

Fall-related risk factors were assessed in person 1 week prior to initiating the intervention. Additionally, at that time, each participant and the exercise instructor were provided with a tablet PC (personal computer), folding chairs, exercise mats, resistance bands, and instructions describing the simple steps necessary to turn on and operate the tablet, facilitating participation in the Web-based telepresence exercise program.

During the intervention period, nutrition guidance and exercise education were provided to all participants once every 4 weeks. Throughout the 12-week intervention period, the participants were encouraged to maintain the same physical activity levels and calorie intake as before participating in the study. During the education sessions, we instructed the participants to inform an instructor holding the education sessions regarding any changes in physical activity levels or nutrition intake. A posttest assessment was performed during the last week of the 12-week program. CG levels of physical activity and nutrition intake were checked every 2 weeks by phone and during the education sessions. An overview of the study design is provided in Figure 1. All data were collected and stored at the Biomedical Research Institute of Seoul National University Hospital in Seoul, Korea.

### Telepresence Platform

**Figure 2:**
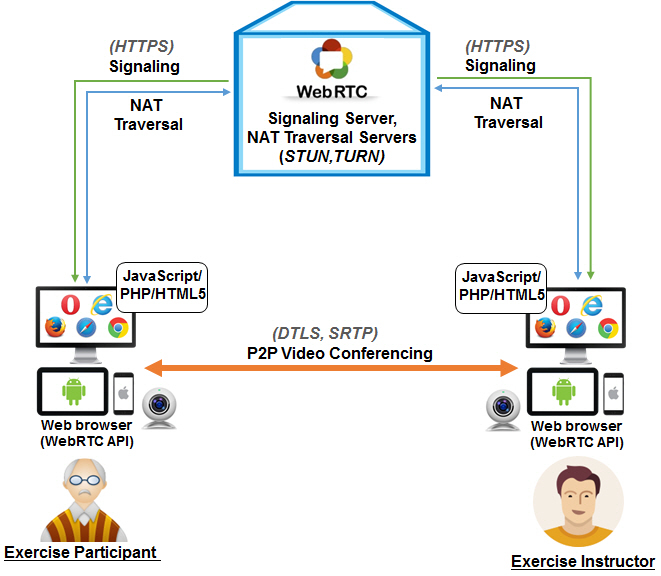
Overview of the telepresence exercise platform using WebRTC technology (API: application programming interface; DTLS: Datagram Transport Layer Security; HTML5: Hypertext Markup Language revision 5; HTTPS: Hypertext Transfer Protocol Secure; ICE: Interactive Connectivity Establishment; NAT: network address translator; P2P: peer-to-peer; PHP: PHP: Hypertext Preprocessor; SRTP: Secure Real-time Transport Protocol; STUN: Session Traversal Utilities for NAT; TURN: Traversal Using Relay NAT; WebRTC: Web Real-Time Communication).

The Web Real-Time Communication (WebRTC) technology enables the telepresence [22] exercise platform to perform real-time voice calling, video chat, and text messaging in the browser without any plug-in software. The telepresence exercise platform consists of Web, Web application, signaling, and network address translator (NAT) traversal server modules that are set up using appropriate programming languages and runtime environments (Hypertext Markup Language revision 5 [HTML5]; PHP: Hypertext Preprocessor [PHP]; JavaScript; Node.js) according to the WebRTC application programming interface (API), as shown in Figure 2. The telepresence operation server system used in this study had the following specifications: Intel Core i3 3.5GHz central processing unit (CPU), 4 GB random-access memory (RAM), Microsoft Windows Server 2012 operating system with an IIS 8 Web server and PHP v5.16 Web application server. The signaling server module enables the telepresence application on each user’s Web browser to set up a call while providing session control messages, media metadata, key data, network data, and error messages. The NAT traversal module is used to cope with gateways or firewalls in real-world networking through the Interactive Connectivity Establishment (ICE), Session Traversal Utilities for NAT (STUN), and Traversal Using Relay NAT protocols (TURN). The actual media and data streaming function in the telepresence application is based on three main WebRTC JavaScript APIs (ie, getUserMedia, RTCPeerConnection, and RTCDataChannel) [23].

We developed the telepresence exercise platform including an operation server system and a website mainly based on WebRTC, which is an open-source technology that works with various operating systems (eg, Windows, Android, iOS) and Web browsers (eg, Chrome, Firefox, Opera, Microsoft Edge). Importantly, WebRTC provides Health Insurance Portability and Accountability Act-compliant security for data transfer (media and signaling) using state-of-the-art encryption standards (Hypertext Transfer Protocol Secure [HTTPS]; Datagram Transport Layer Security [DTLS]; Secure Real-time Transport Protocol [SRTP]) [24]. These properties of WebRTC enhance the usability of this technology in the telepresence service for the elderly. The overall architecture of the telepresence exercise platform is shown in Figure 2.

**Figure 3:**
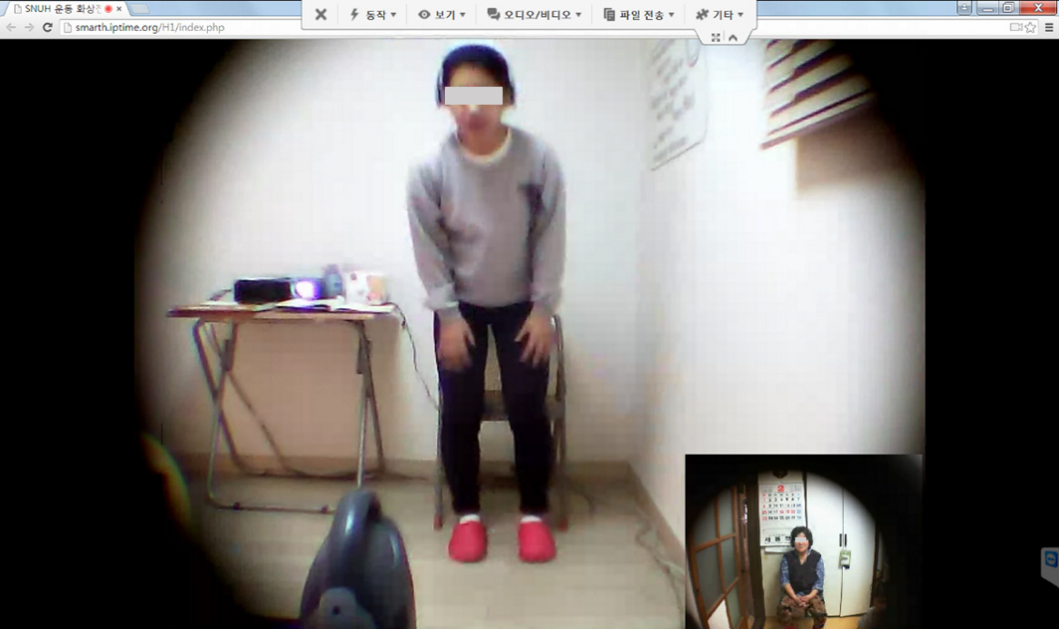
Participant performing telepresence exercises at home using the tablet PC, under the supervision of a remotely located instructor.

An elderly-friendly website was developed for the telepresence exercise sessions (Figure 3). The website was accessed via the Web browser (Google Chrome v26) installed on the tablet PC (X50V2 Plus; Shuttle Inc), which had the following specifications: Intel Atom 1.8 GHz CPU, 2 GB RAM, built-in 15-inch touchscreen, and 2-megapixel webcam with a wide-angle lens. Remote-access software (TeamViewer v8.0) was installed to provide the participants with technical support in operating the tablet. Moreover, broadband network access (10 Mbps) was provided to the instructor and the participants. All items associated with the intervention were provided to the participants and the instructor free of charge.

For the developed telepresence exercise platform, adequate user acceptance testing was conducted by focus groups until March 1, 2015. Throughout the intervention period, no bug fixes or content changes were made to the platform. There was no downtime (planned or unplanned) during the intervention period.

### Telepresence Exercise Program

During the exercise session, the participants turned on the tablet PC, watched the instructor perform the exercise, and followed the instructor’s movements (Figure 3). In order for the instructor to observe the correct movements of the participants, the resistance and balance exercises were performed in the frontal and sagittal planes. The supervised, progressive exercise protocol for IG was based on fall prevention guidelines issued by the Centers for Disease Control and Prevention [25]. The telepresence exercise program included three sessions per week, which took place on non-consecutive days (ie, at intervals of at least 48 hours) for 12 weeks. Each session consisted of a warm-up activity (5 min), main exercise activity (10-30 min), and cool-down activity (5 min). Exercise intensity was controlled based on the Rating of Perceived Exertion (RPE) on the Borg scale [26], as explained below. The warm-up and cool-down activities included stretching and walking in place (9≤RPE≤11), while the main exercise activity consisted of resistance and balance exercises performed using color-coded resistance bands (Thera-Band; Hygenic Corp.) [27] and a chair (11<RPE≤15).

The resistance exercise was performed while sitting on the chair using a yellow (Level 1) band during Weeks 1-4, red (Level 2) band during Weeks 5-8, and green (Level 3) band during Weeks 9-12. This routine, which targeted the major muscle groups in the shoulder, arms, thighs, hips, and calves, included three sets with 8-15 repetitions per set [28]. Specifically, if the RPE was ≤11, the participant was encouraged to increase the number of repetitions performed in the subsequent set (up to a maximum of 15 repetitions); if the RPE was ≥15, the participant was encouraged to decrease the number of repetitions in the subsequent set (down to a minimum of 8 repetitions). The balance exercise included two-legged standing, tandem standing, one-legged standing, semi-tandem standing, tandem walking, turning in a circle around the chair, and exercises such as toe stands, which focused on postural muscle groups. The total exercise time was progressively increased from 20-40 minutes over the course of the intervention period.

To ensure safety and compliance, exercise training was supervised by an instructor who was appropriately trained and had ample experience as an exercise physiologist. The instructor provided one-on-one instructions to each participant, according to the target RPE for each session. All IG participants interacted with the same instructor.

### Outcome Measures

Fall-related physical and psychological factors were examined before and after the intervention. All measurements were performed by an exercise specialist blinded to the group allocation scheme. Body composition and physical function parameters were assessed as physical risk factors for falling, while fall-related self-efficacy (falls efficacy) and fear of falling were assessed as psychological risk factors for falling. The primary outcome for this trial was lower body strength (evaluated in the chair stand test) and balance (evaluated using a balance assessment test). Secondary outcomes included psychological risk factors for falling (falls efficacy, fear of falling).

#### Body Composition

We evaluated body composition in terms of body weight, percentage fat, upper and lower limb muscle mass, and appendicular lean soft tissue (ALST), measured using a dual-energy X-ray absorptiometry device (Lunar Prodigy Advance; GE Healthcare). Total-body skeletal muscle mass (TSM) was calculated using the ALST-based formula proposed by Kim et al [29], as follows: TSM (kg) = 1.13 × ALST – 0.02 × age + 0.61 × sex + 0.97, where sex = 0 for women and 1 for men.

#### Physical Function

The Senior Fitness Test (SFT) and Berg Balance Scale (BBS) were used to evaluate physical functional ability [30]. The SFT consists of the 2-minute step, chair stand, arm curl, chair sit-and-reach, back scratch, and 8-foot up-and-go tests. The BBS is a 14-item test designed to measure the balance of elderly participants by assessing their performance in specific tasks.

In the SFT, the 2-minute step test was used to evaluate cardiorespiratory fitness. The evaluator counted the number of full steps completed by the participant in 2 minutes. A full step was defined as a step performed while raising the knee up to a height corresponding to the midpoint between the patella and the iliac crest. The chair stand test was used to assess the muscle strength and endurance of the lower body, while the arm curl test was used to measure upper body strength. In both these tests, the evaluator counted the number of repetitions performed by the participant in an interval of 30 seconds. The chair sit-and-reach test was used to assess lower body flexibility, while the back-scratch test was used to evaluate upper body flexibility. The 8-foot up-and-go test was used to evaluate agility and dynamic balance. The evaluator noted the time taken by a participant to rise from a seated position, stand up, walk 8 feet, turn, walk back to the chair, and resume the seated position.

In the BBS, each 14-item task is given 4 points on an ordinal scale, ranging from 0 (unable to perform the task) to 4 (performs the task independently). The scores of the 14 tasks are summed to yield a total score ranging from 0-56 points, with a score of 41-56 indicating low risk, 21-40 indicating medium risk, and 0-20 indicating high risk of fall. Higher BBS scores indicate better performance, while scores ≤45 points are predictive of falls.

#### Falls Efficacy

Fall-related self-efficacy, or falls efficacy, reflects an individual’s degree of conviction that they will not fall while performing a certain activity. To measure falls efficacy in community-dwelling Korean women aged >65 years, we used the Korean Falls Efficacy Scale (FES-K) [31], which represents a translated and modified version of the FES-International [32] that takes into account the characteristics of elderly Koreans. The FES-K contains items measuring falls efficacy during activities of daily living and social activities. In our study, falls efficacy was measured on a 4-point Likert scale, and the Cronbach alpha of the FES-K was .87.

#### Fear of Falling

The Fear of Falling Questionnaire (FOFQ) developed by Tideiksaar [33] was used to measure the fear of falling associated with 11 daily activities. Fear of falling was measured on a 4-point Likert scale. In this study, the Cronbach alpha of the FOFQ was .86.

### Statistical Analyses

The fall-related physical and psychological factors were assessed at baseline and at the end of the 12-week study period. An independent *t* test was used to test the homogeneity of physical characteristics between the two groups before the intervention. Two-way repeated-measures analysis of variance (ANOVA) was performed to account for the effect of group (IG and CG), time (pre- and posttest), and interaction between group and time. When there were significant interaction effects, a paired *t* test was used to assess within-group differences between pre- and posttest measurements. The significance threshold was *P*<.05 for all tests. All statistical analyses were performed using SPSS (version 22.0; IBM Corp).

## Results

### Participant Characteristics

The homogeneity test for physical characteristics and dependent variables showed that, at baseline, IG and CG did not differ significantly regarding age, body weight, body height, percentage fat, ALST, or TSM (Table 1).

**Table 1:** Baseline physical characteristics.

Characteristics	Intervention group (n=10), mean (SD)	Control group (n=13), mean (SD)	*P* value^a^
Age years	78.10 (5.66)	81.54 (5.07)	.14
Weight, kg	56.18 (6.54)	55.70 (9.03)	.47
Height, cm	150.30 (4.52)	148.76 (5.30)	.89
Percent fat	33.53 (6.48)	31.89 (10.21)	.66
Appendicular lean soft tissue, kg	14.22 (2.03)	13.97 (1.11)	.71
Total skeletal muscle mass, kg	15.49 (2.31)	15.14 (1.23)	.65

^a^*P* values represent homogeneity and were obtained using an independent *t* test.

**Table 2:** Changes in body composition. *Before* and *After* refer to measurements taken before and after the 12-week intervention period, respectively.

Variable	Intervention group (n=10), mean (SD)		Control group (n=13), mean (SD)		Interaction, *P* value^a^
	Before	After	Before	After	
Weight, kg	56.18 (6.45)	57.39 (7.12)	55.70 (9.03)	55.86 (9.13)	.18
Percent fat	33.53 (6.48)	34.36 (6.59)	31.89 (10.21)	32.33 (9.85)	.52
Upper limb mass, kg	3.63 (0.50)	3.69 (0.78)	3.43 (0.40)	3.31 (0.36)	.10
Lower limb mass, kg	10.58 (1.55)	10.96 (1.82)	10.53 (0.94)	10.37 (0.80)	.04
Appendicular lean soft tissue, kg	14.22 (2.03)	14.65 (2.56)	13.97 (1.11)	13.68 (0.88)	.03
Total skeletal muscle mass, kg	15.49 (2.31)	15.98 (2.89)	15.14 (1.23)	14.82 (0.99)	.03

^a^The interaction effect between time and group was evaluated in terms of the *P* value obtained using two-way repeated-measures analysis of variance.

### Body Composition

Table 2 provides an overview of the changes in body composition between baseline (before intervention) and the end of the 12-week intervention period. Significant interaction effects between group and time were seen for lower limb muscle mass (*P*=.04), ALST (*P*=.03), and TSM (*P*=.03). Post hoc analyses revealed no significant change in either group, although IG participants showed a tendency of increase in lower limb muscle mass, ALST, and TSM. No significant changes were noted for body weight, percentage fat, or upper limb muscle mass.

### Physical Function

Table 3 provides an overview of the changes in physical function between baseline (before intervention) and the end of the 12-week intervention period. Two-way repeated-measures ANOVA revealed a significant interaction effect between group and time regarding the scores of the chair stand test (*P*<.001) and BBS (*P*=.03). However, there were no changes in the scores for the 2-minute step, arm curl, back scratch, chair sit-and-reach, or 8-foot up-and-go tests. With respect to the chair stand test, which measures lower body strength, a significant increase was found for both IG (*P*<.001) and CG (*P*=.04) participants. Additionally, BBS scores showed a significantly larger increase among IG participants than among CG participants (*P*=.02).

### Falls Efficacy and Fear of Falling

Table 4 provides an overview of the change in scores for falls efficacy and fear of falling between baseline (before intervention) and the end of the 12-week intervention period. Two-way repeated-measures ANOVA revealed significant interaction effect between group and time regarding the fear of falling (*P*=.009). Scores for fear of falling showed a significantly larger decrease among IG participants than among CG participants (*P*=.008). There were no changes in falls efficacy.

**Table 3:** Changes in physical function. *Before* and *After* refer to measurements taken before and after the 12-week intervention period, respectively.

Test	Intervention group (n=10), mean (SD)		Control group (n=13), mean (SD)		Interaction, *P* value^a^
	Before	After	Before	After	
2-min step, steps	54.40 (31.26)	67.00 (41.33)	68.92 (24.22)	64.76 (29.92)	.09
Arm curl reps	12.60 (4.62)	23.70 (5.47)	15.15 (3.55)	23.53 (4.19)	.18
Chair stands	11.00 (4.64)	19.20 (5.99)^b^	13.00 (2.61)	14.15 (2.70)^c^	<.001
Back scratch, cm	-13.60 (11.76)	-15.20 (10.46)	-14.23 (9.01)	-13.15 (8.79)	.31
Chair sit-and-reach, cm	14.10 (10.83)	11.10 (11.42)	16.30 (8.05)	11.92 (8.85)	.73
8-foot up-and-go, seconds	9.55 (4.03)	8.90 (2.76)	8.27 (2.27)	8.52 (1.75)	.40
Berg Balance Scale score	43.00 (6.49)	44.30 (6.32)^e^	44.69 (3.49)	43.84 (3.57)	.03

^a^The interaction effect between time and group was evaluated in terms of the *P* value obtained using two-way repeated-measures analysis of variance.

^b^*P*<.001.

^c^*P*<.05. Values indicate a significant difference between pre- and posttest measurements (ie, Before vs After for the same group).

**Table 4:** Changes in psychological risk factors for falling. *Before* and *After* refer to measurements taken before and after the 12-week intervention period, respectively.

Assessment tool		Intervention group (n=10), mean (SD)		Control group (n=13), mean (SD)		Interaction, *P* value^a^
		Before	After	Before	After	
**Falls efficacy**						
	Activities of daily living	15.80 (3.93)	15.90 (5.23)	16.53 (3.17)	17.23 (2.68)	.55
	Social activities	13.70 (2.31)	14.60 (1.71)	13.23 (2.91)	13.53 (3.45)	.51
Fear of falling		30.50 (7.60)	28.40 (7.74)^b^	29.46 (4.68)	29.53 (4.99)	.009

^a^The interaction effect between time and group was evaluated in terms of the *P* value obtained using two-way repeated-measures analysis of variance.

^b^*P*<.01 indicates a significant difference between pre- and posttest measurements (ie, Before vs After for the same group).

## Discussion

### Principal Results

The objective of this study was to develop a novel telepresence exercise platform based on WebRTC technology, as well as a suitable telepresence exercise program, and to investigate the effect of such a home-based telepresence program (ie, with real-time supervision and feedback from a specialized instructor) on fall-related risk factors in elderly women. The main finding was that a 12-week telepresence program involving progressive exercise can be effective for enhancing physical performance (chair stand test score), improving balance (BBS score), and reducing fear of falling in elderly women at risk of falls.

### Comparison With Prior Work

In the elderly population, falls are an important public health concern, and exercise interventions are essential to fall prevention [5,34]. Decrease in muscle function has been identified as an independent predictor of disability and death [35], and reduced strength in the lower limbs has a detrimental effect on gait speed, balance, and the ability to rise from a chair [36,37]. Increasing physical performance and balance ability is thus an important strategy to improve social participation and quality of life, especially among elderly individuals with high risk of falling. The benefits are reflected as improved ability to perform activities of daily living, reduced fear of falling [38], enhanced physical activity parameters such as walking speed and stair climbing ability, increased muscle fiber cross-sectional area, and increased muscle strength [39]. These benefits are thought to be due to muscle nerve stimulation by regular exercise, and activation of the somatosensory system as a result of lower body bending and extension.

Recent studies have investigated the effectiveness of home-based exercises in reducing fall-related risk factors in the elderly population. Campbell et al [40] reported that home exercises for muscle strength and balance increased physical function and reduced the incidence of falls and injuries among very elderly women (≥80 years). Yates and Dunnagan [41] found that a 10-week home-based exercise program induced significant improvement in lower extremity strength and balance among rural community-dwelling older adults, which is similar to the findings of our study. Overall, these studies support the use of home-based exercises to reduce the risk of falls. Wu and Keyes [42] reported that a home-based Tai Chi exercise program involving videoconferencing provided an increased sense of balance among elderly participants, which is also similar to the findings of our study, although it should be mentioned that the aforementioned study was limited by the lack of a control group.

The findings of our study are contrary to those of Sosnoff et al [43], who found that a 12-week home-based exercise program provided no significant improvement in lower limb strength or BBS scores among older adults with multiple sclerosis. The discrepancy between these findings is likely due to differences in the intervention methods applied in the two studies. Namely, the participants of our study used a face-to-face method that involved looking at the computer monitor and following the instructor’s movements at home, while the participants in the previous study performed exercises at home by consulting a distributed manual and did not receive immediate feedback about their movements. These differences emphasize the importance of direct interactions with the instructors.

Among the elderly, decreased ability to maintain balance translates into an increased incidence rate of falls. Moreover, elderly individuals who are unable to maintain balance often develop a fear of falling, which results in reduced levels of physical activity and reluctance to lead an independent life [7]. Fear of falling, which represents a psychological risk factor related to falls, is defined as a lasting concern regarding falling, which leads an individual to avoid activities that they are otherwise fully capable of performing [44]. Fear of falling can range from a healthy concern about avoiding risky situations, such as navigating an icy sidewalk, to a more severe and disabling anxiety about falling, which can negatively affect an older individual’s independence [45]. It is currently accepted that fear of falling can influence the incidence of falls, which suggests that reducing the fear of falling is critical to reducing the incidence rate of falls. The elderly can reduce their fear of falling by exercising regularly and increasing their fall-related self-efficacy (ie, conviction that they will not fall).

In this study, we found that telepresence exercise provided a significant reduction in the fear of falling, which is similar to the observations reported by Liu and So [46] regarding the effect of Tai Chi exercises in elderly individuals living in a health facility. This finding is, however, contrary to that of Schoenfelder [47], who reported that a 3-month supervised program of ankle strengthening exercises and walking did not reduce the fear of falling in older adults. The discrepancy between these findings may be attributed to the fact that the participants in our study were older and had a higher risk of falls. In other words, the participants had a considerably high fear of falling prior to the study and hence demonstrated a greater decrease in their fear of falling after completing the exercise program, according to the principle of initial values [48], which is one of the key principles of exercise training.

It is important to note that the women who participated in our telepresence exercise program interacted with their instructor on a one-on-one and face-to-face basis, rather than performing simple exercises and following an unsupervised education program, suggesting that the decrease in their fear of falling might be related to positive changes induced by real-time and sustained interactions. Several studies highlighted the fact that there are limits to the effect of interventions aiming to prevent falls in very elderly people (≥80 years) and proposed that this population will benefit more from an in-home approach accompanied with one-on-one management, rather than from a group exercise program [7,18].

In our study, there was no statistically significant difference between the intervention and control groups regarding fall efficacy, which was measured with respect to activities of daily living and social activities. While both groups showed an increase in the falls efficacy with respect to social activities, the intervention group exhibited a numerically greater increase. We speculate that exercise training can be expected to lead to an increase in social activity participation among elderly individuals. The increase in participation in activities of daily living and social activities among women in the control group may have been due to an increase in confidence in performing movements involved in these activities, which may have been promoted by nutrition guidance and exercise education.

Our findings offer objective evidence that an at-home exercise routine with real-time feedback represents an effective intervention for improving balance and reducing fear of falling among the elderly. It is important to note that the value of telepresence exercise is not limited to its effectiveness in improving fall-related risk factors among elderly women at risk of falls and provides additional advantages associated with its design. First, the developed telepresence exercise platform appears to be optimally suited for elderly participants, as both the tablet and the website were very user-friendly and allowed the elderly participants to operate them with a minimum amount of effort. Second, the one-on-one exercise approach enabled participants to receive real-time feedback and engage directly with the instructor, which is typically difficult during group exercises. Supervision and timely feedback regarding the exercises not only improved the effectiveness of training (versus that of unsupervised training) but was also important to ensure the safety of the participants, who were elderly. Third, the study was successfully conducted during a period when the risk of falls among the elderly was particularly high owing to cold temperatures and heavy snowfall. However, the weather conditions did not affect the implementation of the telepresence exercise program (attendance or compliance), as such exercises were conducted in the safety of the participants’ homes.

### Limitations

This study has several limitations. First, the sample size was relatively small, which precludes generalization of our findings. Second, a cost-benefit analysis for the telepresence exercise platform service was not conducted. Third, follow-up evaluation after 12 weeks is needed to confirm the long-term effects of telepresence exercise. Future studies should include a larger number of participants, a quantitative cost-effectiveness analysis, and a long-term effectiveness analysis.

### Conclusions

We found that home-based training using our telepresence exercise platform and remote supervision by a specialist improved fall-related physical and psychological factors in home-dwelling elderly women. Telepresence exercise can be used in a broad range of populations that need remote training, including patients with disabilities and older adults with reduced mobility.

## Appendix

Multimedia Appendix 1 CONSORT‐EHEALTH checklist (V 1.6.1).

## Abbreviations

ALST: appendicular lean soft tissue
ANOVA: analysis of variance
API: application programming interface
BBS: Berg Balance Scale
CG: control group
FES-K: Korean Falls Efficacy Scale
FOFQ: Fear of Falling Questionnaire
IG: intervention group
NAT: network address translator
PHP: PHP: Hypertext Preprocessor
RPE: rating of perceived exertion
SFT: Senior Fitness Test
TSM: total-body skeletal muscle mass
WebRTC: Web Real-Time Communication
